# How Diphtheria Antitoxin Is Made

**Published:** 1913-04

**Authors:** 


### How Diphtheria Antitoxin is Made.

In initiating the immunizing treatment of the horse, a first dose of about 1000 units of diphtheria antitoxin is injected subcutaneously, this being followed by a relatively small dose of the specific toxin. After several days, when the reaction and fever have abated, a still larger dose of toxin is injected; and the treatment is continued in this manner for at least two months. At the end of this period the horse in all probability will be conditioned to produce a considerable amount of antitoxin, although in a large percentage only a very small quantity is yielded, such horses proving utterly worthless for this purpose. A well-adapted horse will show from 300 to 900 units per c.c.

When the animal under treatment shows a sufficient number of units per c.c. to warrant bleeding, its blood is drawn off through a cannula inserted into the jugular vein. By means of a rubber tube attached to the cannula the fluid is conducted into bottles containing a solution of sodium citrate, which prevents clotting. As much as 12 liters at a time may be drawn without weakening a horse to any extent.

The bottles containing the blood are allowed to stand for about twenty-four hours, during which time the red and the white cells and all floating material, because of slightly greater specific gravity, gradually settle to the bottom, leaving the plasma with its contained antitoxic bodies at the top, whence they may be decanted off.

### THE REFINING AND CONCENTRATING OF THE SERUM.

In the early days of antitoxin therapy the crude plasma, containing all of the irritating and rash-producing properties of horse-serum, was injected. A comparatively large volume was required for a dose, as at that time it was not often that a serum containing as high as 500 units per cubic centimeter was procured from a horse. Even at this rate, 2 c.c. of fluid often was required for an ordinary immunizing dose, while for a curative dose often as much as 20 c.c., or even more, was necessary. Hence, the injection of antitoxic serum was really nothing less than heroic treatment.

No great improvement was made upon this state of affairs until the year 1904, when Dr. Gibson, of the New York Department of Health, announced his method of concentrating and refining the antitoxic serum by precipitating out the antitoxic globulin. As a result of his experiments, he was able to remove from the serum a large percentage of the non-antitoxic bodies. Hence, the volume being greatly diminished and most of the irritating properties removed, the severity of the injection was greatly reduced. The method of Gibson has since been markedly improved upon by Dr. Banzhaf, of the same laboratories.

Probably the most widely used method of refining and concentrating antitoxic plasma is the following modification of the Gibson method:

The plasma, after having been drawn off from the red and white cells, is heated at a constant temperature of 56° C. for fifteen hours and then diluted with one-third of its volume of water. By means of ammonium sulphate, the globulin, anti-toxic and nonantitoxic, is precipitated. On filtering through paper, this precipitate is obtained separately from the albumin and various other soluble constituents. It is then brought into solution again by means of sodium chloride, when the antitoxic globulin may be precipitated with dilute acetic acid. After another filtration, the isolated antitoxic globulin is obtained as a precipitate on the papers.

The filter-papers with this precipitate upon them are placed in a press in pairs, face to face with an intervening layer of absorbing paper between each pair, and the water, with a large percentage of all soluble material, is pressed out. The pressing completed, all salts are removed by dialysis. This antitoxic globulin solution is then brought up to the normal salt constituency of the blood.

After dialysis, the antitoxin is placed on ice for at least two weeks, when it is filtered through paper pulp to remove whatever insoluble matter it may contain, and then through a Berkfield clay filter, to eliminate all bacteria. We now have the antitoxic globulin itself, almost entirely free from extraneous matter.

The dread of rashes, serum sickness, and so on, is gone forever. Nor is this the only improvement, for the volume (now tremendously reduced) is of greatest importance. Where previously 10 c.c. or more was required for a dose of 5000 units, today the physician needs to inject only 2 c.c., or even less; while the danger from ill-effects is reduced to practically nothing.—Abstract of an article by Arthur M. Slee, Immunologist at the Slee Laboratories, Swiftwater, Pa., republished from the *American Journal of Clinical Medicine*, January, 1913.

---

Dr. A: I operated on him for appendicitis.

Dr. B: Did you? What was the matter with him?—*Ex.*

